# Staphylococcus lugdunensis Infective Endocarditis With Mitral Valve Vegetations in a Hemodialysis Patient With Recurrent Arteriovenous Fistula Cannulation: A Case Report

**DOI:** 10.7759/cureus.39853

**Published:** 2023-06-01

**Authors:** Aliaa Mousa, Ahmed Ghazy, Tigran Kakhktsyan, Kateryna Chepenko, Kristopher Young

**Affiliations:** 1 Internal Medicine, Capital Health Regional Medical Center, Trenton, USA; 2 Cardiology, Capital Health Regional Medical Center, Trenton, USA

**Keywords:** infective endocarditis, vancomycin infusion, mitral valve vegetation, hemodialysis access, staphylococcus lugdunensis

## Abstract

Infective endocarditis is a severe medical condition that occurs when the endocardium of the heart gets infected by different microorganisms, including coagulase-negative staphylococci such as *Staphylococcus lugdunensis*. The source of infection is often related to procedures done in the groin area, such as femoral catheterization for cardiac catheterization, vasectomy, or central line placement in an already infected mitral or aortic valve. Herein, we are discussing a case of a 55-year-old female with a past medical history of end-stage renal disease on hemodialysis with a history of recurrent cannulation of her arteriovenous (AV) fistula. She presented with fever, myalgia, and generalized weakness, and was later found to have *Staphylococcus lugdunensis* bacteremia and infective endocarditis with mitral valve vegetations, for which the patient was transferred to the mitral valve specialized center for mitral valve replacement. This case acts as a reminder to consider recurrent cannulation of the AV fistula as one of the potential ports of entry of *Staphylococcus lugdunensis* to the body.

## Introduction

Infective endocarditis (IE) caused by *Staphylococcus lugdunensis* with mitral valve vegetations is a rare condition and has been reported infrequently since it was first identified as a causative pathogen of IE in 1988. Studies conducted in 2018 [1] suggest that groin manipulations or unknown entry are the typical access points, leading to vegetation on the left-sided heart valves [2], predominantly the mitral valve. This condition requires medical treatment with antibiotics and definitive surgical intervention [3].

According to a study published in the Journal of the American Society of Nephrology, the incidence of IE in end-stage renal disease patients on hemodialysis is 10- to 30-fold higher than in the general population. Furthermore, the most common causative organisms in hemodialysis-associated IE are *Staphylococcus aureus* and coagulase-negative staphylococci. However, *S. lugdunensis*, which is part of the coagulase-negative staphylococci group, has recently been identified as a rare but increasingly recognized cause of hemodialysis-associated IE [4]. The present case highlights the relationship between repeated cannulation of hemodialysis access in a naive mitral valve and the development of IE in a patient with end-stage renal disease.

## Case presentation

**Figure 1:**
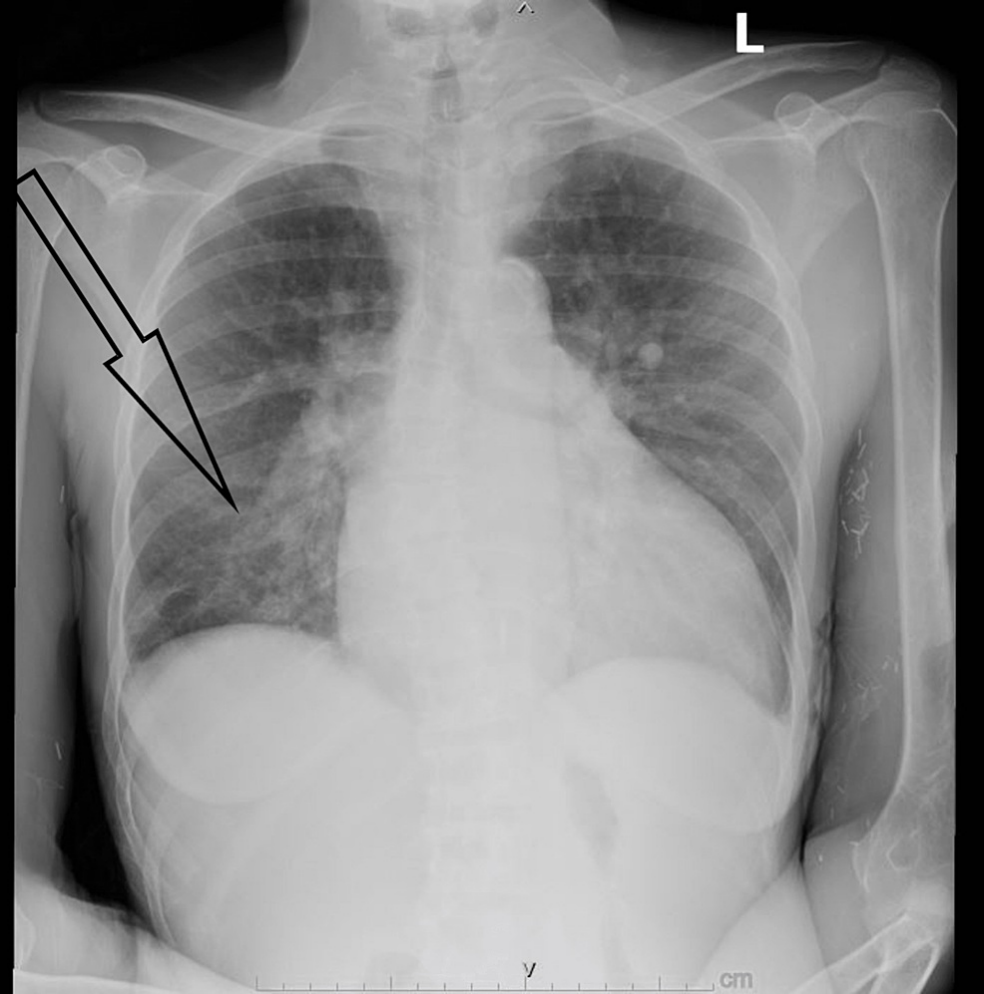
Chest X-ray showing right lower lobe pneumonia

A 55-year-old woman with a past medical history of failed kidney transplant and end-stage renal disease requiring hemodialysis since 2021, hypertension, diabetes, chronic obstructive pulmonary disease, sleep apnea, and jugular vein thrombosis presented to the hospital due to worsening shortness of breath, productive cough, myalgia, arthralgia, generalized body aches, chills, sweats, and fever over the past two weeks. She had to interrupt her hemodialysis session earlier that day due to worsening shortness of breath. On physical examination, she appeared fatigued and chronically ill, with no jugular vein distention, a 3/6 apical systolic murmur, and a left upper extremity arteriovenous fistula with good bruit and thrill. Her vital signs showed a temperature of 39.3°C, heart rate of 112 bpm, respiratory rate of 18 breaths per minute, blood pressure of 160/80 mmHg, and oxygen saturation of 96% on room air. Basic blood work showed an elevated white blood cell count of 12,000/uL (Table 1), and a chest X-ray (Figure 1) revealed a ground-glass opacity at the right lung base, suggestive of pneumonia. A first set of blood cultures was drawn before starting broad-spectrum antibiotics. Later, blood cultures showed growth of *Staphylococcus lugdunensis, *isolated from aerobic and anaerobic bottles, which was sensitive to vancomycin. Infectious disease consultation was requested, upon which vancomycin goal trough 15-20 was continued and cefepime was stopped, repeat two sets of blood cultures were ordered to assure clearance of bacteremia, and the source of infection was suspected to be recurrent cannulation of her fistula as the patient at the time of presentation had left upper extremity fistula, that is currently used for hemodialysis but she also used to have an old fistula on the right upper extremity that developed pseudoaneurysm that was removed. The patient also denied any recent history of dental and groin procedures. Two-dimensional echocardiography for initial evaluation of infective endocarditis was done, which showed normal left ventricular size with hypertrophy and normal systolic function, severe left atrial dilatation, moderate mitral sclerosis, and a small to moderate size vegetation on the posterior leaflet with no significant mitral regurgitation. At that point, cardiology was consulted for further assessment. A transesophageal echocardiogram (TEE) was done (Videos 1, 2), which showed a large, highly mobile vegetation on the posterior leaflet of the mitral valve, measuring 23 mm in length and 10 mm in width. Due to persistent bacteremia with the repeat blood cultures that were still positive for *Staphylococcus lugdunensis*, and the size and risk of embolization, the patient was recommended for transfer to a high-volume mitral valve center for surgical evaluation. Over there, the patient had open heart surgery within 72 hours and mechanical mitral valve replacement, and then was started on intravenous heparin to be bridged to oral warfarin. On the sixth postoperative day, the patient was discharged on oral warfarin 6 mg with a goal of international normalized ratio of 2.5-3.5, and six weeks of vancomycin on hemodialysis days.

**Table 1:** Basic blood workup

Tests	Results	Normal range
White blood cell count	12.14 x10^3/mcL	4 to 10 x10^3/mcL
Hemoglobin	9.7 g/dL	11.2 to 15.7 g/dL
Red blood cell count	3.61 x10^6/mcL	3.9 to 5.2 x10^6/mcL
Platelets	119	150 to 400
Sodium	131 mmol/L	137 to 145 mmol/L
Potassium	4.1 mmol/L	3.5 to 5.1 mmol/L
Chloride	93 mmol/L	98 to 107 mmol/L
Bicarbonate	30 mmol/L	22 to 30 mmol/L
Serum creatinine	5.72 mg/dl	0.52 to 1.04 mg/dl
Blood urea nitrogen	31 mg/dl	7 to 17 mg/dl
Alkaline phosphatase	123 U/L	38 to 126 U/L
Aspartate transaminase	20 U/L	14 to 36 U/L
Alanine transaminase	11 U/L	0 to 34 U/L
Albumin	2.9 g/dl	3.5 to 5.0 g/dl
Lactic acid	0.9 mg/dl	0.7 to 1.9 mmol/L
Estimated glomerular filtration rate	8	NL low >60
Phosphorus	7.2 mg/dl	2.4to 4.5 mg/dl

**Video 1:** Transesophageal echocardiogram (black and white, parasternal window) showing vegetation on the posterior leaflet of the mitral valve

**Video 2:** Three-dimensional transesophageal echocardiogram showing mobile vegetation (23 mm x 10 mm) on the posterior leaflet of the mitral valve

## Discussion

*Staphylococcus lugdunensis* is a coagulase-negative staphylococcus that has emerged as an important pathogen causing various infections, including IE. It is considered an aggressive pathogen, especially in chronic immunocompromised personnel with a high potential for morbidity and mortality. In a study published in the Journal of Clinical Microbiology [2], *S. lugdunensis* was found to be the fourth most common cause of IE, after *S. aureus*, viridans streptococci, and enterococci [3]. The infection can occur in both native and prosthetic valves and can be associated with large vegetations, with a higher prevalence for mitral valves than other valves [1].

Patients with intravascular catheters for hemodialysis, implanted ports for chemotherapy, vascular prostheses, or intracardiac devices account for approximately 10% of IE of the right heart. A study conducted by Jiang et al. in China, involving 412 patients with IE who required surgical treatment over a 10-year period, reported that 35 patients (8.5%) had right-sided IE. Among them, one patient was receiving hemodialysis, accounting for 2.8% of the total cases. This highlights the relatively low occurrence of right-sided IE in hemodialysis patients. Similarly, in a study by Musci et al. in Germany spanning 20 years, out of 57 patients with isolated right heart IE, three were undergoing hemodialysis, representing 5.2% of the cases. These findings further support the observation that right heart IE is relatively uncommon in hemodialysis patients, and *S. aureus* still plays the main role of the infective pathogen [5].

While most reported cases of *Staphylococcus* *lugdunensis* IE have indicated that the source of infection was related to groin manipulation [1], in the case we are discussing, it is rarely reported for the port of entry to be repeated manipulation of a fistula in a patient with end-stage renal disease who is undergoing hemodialysis.

Mitral valve vegetations are a hallmark of IE and are usually the result of bacterial colonization of the valve surface. The same study [2] also showed the presence of vegetation increases the risk of complications, including embolization, valve destruction, IE complicated with heart block, annular or aortic abscess, persistent bacteremia lasting for five to seven days after initiation of antibiotics, and heart failure. The size of the vegetation is an important factor that determines the risk of complications. Vegetations larger than 10 mm in diameter are associated with a higher risk of embolization. The same complications mentioned above are absolute indications for urgent surgery regardless of the completion of a course of antibiotics [6].

In the case of our patient, the presence of a large, highly mobile vegetation on the posterior leaflet of the mitral valve, measuring 23 mm in length and 10 mm in width, raises concerns about the risk of embolization. The decision to transfer the patient to a high-volume mitral valve center for surgical evaluation is appropriate given the size and mobility of the vegetation.

The identification of *Staphylococcus lugdunensis* as the causative agent of IE is significant because of its high virulence and resistance to some antibiotics [7]. Vancomycin is the drug of choice for treating staphylococcal endocarditis, and in this case, the sensitivity of the organism to vancomycin was high. However, close monitoring of the patient is necessary, as *Staphylococcus lugdunensis* has been associated with a high rate of complications and mortality in IE [8].

The mechanisms by which *S. lugdunensis* causes mitral valve vegetations are not well understood, but it is thought to involve the bacteria's ability to form biofilms on the valve surface [9]. Biofilms are communities of bacteria that are encased in a protective matrix and are more resistant to antibiotics than individual bacteria. The formation of biofilms on the mitral valve can cause damage to the valve tissue and promote the formation of vegetation. Biofilms are known to be highly resistant to antibiotics and can provide a protective environment for bacteria to grow and thrive.

## Conclusions

The case of our patient highlights the potential severity of IE caused by *Staphylococcus lugdunensis* and emphasizes the importance of early diagnosis and appropriate treatment. The port of entry might be an unusual site here, which is a repeatedly cannulated arteriovenous hemodialysis fistula. The presence of large mitral valve vegetation increases the risk of complications, and surgical evaluation may be necessary to prevent embolization and further damage to the valve.
